# miRNA-27b Targets Vascular Endothelial Growth Factor C to Inhibit Tumor Progression and Angiogenesis in Colorectal Cancer

**DOI:** 10.1371/journal.pone.0060687

**Published:** 2013-04-12

**Authors:** Jun Ye, Xianguo Wu, Dang Wu, Pin Wu, Chao Ni, Zhigang Zhang, Zhigang Chen, Fuming Qiu, Jinghong Xu, Jian Huang

**Affiliations:** Cancer Institute (Key Laboratory of Cancer Prevention and Intervention, China National Ministry of Education, Key Laboratory of Molecular Biology in Medical Sciences, Zhejiang Province, China), Second Affiliated Hospital, Zhejiang University School of Medicine, Hangzhou, China; Vanderbilt University Medical Center, United States of America

## Abstract

Colorectal cancer (CRC) is one of the most prevalent cancers globally and is one of the leading causes of cancer-related deaths due to therapy resistance and metastasis. Understanding the mechanism underlying colorectal carcinogenesis is essential for the diagnosis and treatment of CRC. microRNAs (miRNAs) can act as either oncogenes or tumor suppressors in many cancers. A tumor suppressor role for miR-27b has recently been reported in neuroblastoma, while no information about miR-27b in CRC is available. In this study, we demonstrated that miR-27b expression is decreased in most CRC tissues and determined that overexpression of miR-27b represses CRC cell proliferation, colony formation and tumor growth *in vitro* and *in vivo*. We identified vascular endothelial growth factor C (VEGFC) as a novel target gene of miR-27b and determined that miR-27b functioned as an inhibitor of tumor progression and angiogenesis through targeting VEGFC in CRC. We further determined that DNA hypermethylation of miR-27b CpG islands decreases miR-27b expression. In summary, an anti-tumor role for miR-27b and its novel target VEGFC *in vivo* could lead to tumor necrosis and provide a rationale for developing miR-27b as a therapeutic agent.

## Introduction

Colorectal cancer (CRC) ranks as the third most prevalent cancer worldwide. Despite the clinical implementation of numerous therapeutic strategies, it remains a leading causes of cancer-related deaths due to therapy resistance and metastasis [1]–[3]. Therefore, understanding the mechanism underlying colorectal carcinogenesis is essential for diagnosis and treatment of CRC. Interactions between tumors and the stroma are recognized as critical components of tumor progression in CRC [4]. More recently, the evidence indicating that chemokines produced within the tumor microenvironment such as vascular endothelial growth factor (VEGF), fibroblast growth factor (FGF), and platelet-derived growth factor (PDGF) play a crucial role in the pathogenesis of CRC is increasing [5], [6].

microRNAs (miRNAs) are a class of small, endogenous, non-coding RNA, which play important roles in the regulation of target genes by complementary pairing in the mRNA 3′ untranslated region (3′UTR) that leads to translational repression or mRNA degradation [7]. miRNAs are known to function in diverse biological processes including development, cell proliferation, differentiation, apoptosis, and cancer initiation or progression [8], [9]. In cancer, miRNAs can act as either an oncogene or a tumor suppressor, as evidenced by miR-130b promoting liver cancer stem cells (CSCs) growth and self-renewal via targeting TP53INP1 [10], miR-34a inhibiting prostate cancer metastasis by directly repressing CD44 [11], and miR-7 inhibiting tumor growth and metastasis by affecting the the phosphoinositoide-3 kinase (PI3K)/AKT pathway in hepatocellular carcinoma [12]. These results suggest that it is of pivotal importance to clarify miRNA functions and regulatory circuits to formulate therapeutic strategies.

We hypothesize that molecular differences between CSCs and differentiated cancer cells may identify a key molecule in tumor growth and progression, and in this study, investigated differences in miRNA expression between CSCs and differentiated CRC cells using miRNA microarrays. We found that miR-27b expression is substantially decreased in CSC-like cells and in CRC tissues. miR-27b is located on chromosome 9 and has been shown to function as a tumor suppressor in neuroblastoma via targeting the peroxisome proliferator-activated receptor γ (PPARγ) [13]. It has also been reported that miR-27b can act as an angiogenic switch by promoting endothelial tip cell fate and sprouting [14], [15]. However, the specific functions and potential targets of miR-27b in CRC cells are unexplored. We confirmed that vascular endothelial growth factor C (VEGFC), which plays a role in tumor progression, is a novel target of miR-27b. A large number of clinical studies have shown that increasing expression of VEGFC in primary tumors correlated with enhanced dissemination of tumor cells to regional lymph nodes in a variety of human carcinomas [16]–[18]. Recently, the regulatory role of VEGFC in initiating and potentiating neo-angiogenesis had been uncovered [19]. We discovered that miR-27b could block CRC cell proliferation, colony formation and tumor growth and that it functions as an angiogenesis inhibitor by targeting VEGFC and down-regulating DNA hypermethylation. Understanding the mechanisms by which miR-27b inhibits tumor growth and angiogenesis establishes a strong rationale for its development as a therapeutic anti-tumor agent.

## Materials and Methods

### Ethics Statement

This research was approved by the Institutional Review Boards of Second Affiliated Hospital of Zhejiang University School of Medicine. All participants gave written consent of their information to be stored in the hospital database and used for research.

All Animal works had been conducted according to relevant national and international guidelines. This research was approved by the Institutional Review Boards of Second Affiliated Hospital of Zhejiang University School of Medicine.

### Cell Lines

The human colorectal cancer cell lines, SW620, SW480, RKO, HT29 and 293T were purchased from the cell bank at the China Academy of Medical Science (China). SW620 and SW480 cells were cultured in Leibovitz L15 medium (Gibco, Carlsbad, CA, USA) supplemented with 10% fetal bovine serum (FBS, Gibco). RKO, HT29 and 293T cells were cultured in RPMI 1640 medium (Gibco) supplemented with 10% FBS. All cells were maintained at 37°C in a humidified 5% CO_2_ atmosphere.

### miRNA Expression Microarray Analysis

Total RNA was isolated from CD133^+^ and CD133^−^ CRC cells using TRIzol® reagent (Invitrogen, Carlsbad, CA, USA) according to the manufacturer’s protocol. The quantity and the quality of RNA were evaluated using a Nanodrop spectrophotometer (Thermo scientific, Worcester, MA, USA). The miRNA expression profile of each sample was assessed using an Affymetrix miRNA array (Affymetrix, Santa Clara, CA, USA).

### Quantitative PCR Analysis

Total RNA from cell lines, fresh CRC tissues or xenograft tissues was isolated using TRIzol® reagent (Invitrogen). Total RNA from paraffin-embedded tissues was isolated by RecoverAll™ Total Nucleic Acid Isolation Kit (Applied Biosystems, Foster City, CA, USA) and treated with RNase-free DNase I (Qiagen, Valencia, CA, USA) according to the manufacturer’s instructions. The quantity and the quality of RNA were evaluated using a Nanodrop spectrophotometer. TaqMan miRNA expression assays (Applied Biosystems) were used to quantify miRNA expression using the StepOnePlus™ system (Applied Biosystems). All samples were run in triplicate, and miR-27b levels in each sample were normalized to that of U6.

### Proliferation Assay

Cells were seeded at a density of 3×10^3^ cells per well in a 96-well plate containing 0.2-ml Leibovitz L15 medium with 10% FBS. MTS (3-[4, 5-dimethylthiazol-2-yl]-5-[3-carboxymethoxyphenyl]-2-[4-sulfophenyl]-2H-tetrazolium salt) reagent (Promega, Madison, WI, USA) (20 µl) was added to each well and the cells were incubated at 37°C for 4 h. The absorbance values were measured at 490 nm on a microplate reader (Bio-Rad, Hercules, CA, USA) and assessed continuously for 7 days.

### Soft-agar Colony Assay

Cells were seeded at a density of 300 per well on the top layer of 0.3% low-melting agarose (Sigma, St Louis, MO, USA) in 12-well plates with a bottom layer of 0.5% agarose in Leibovitz L15 medium containing 10% FBS. After incubation at 37°C in a humidified 5% CO_2_ atmosphere incubator for 2 weeks, colonies containing 20 cells were visualized under an inverted microscope and counted.

### Tumorigenesis Assay

Cells suspended in 100-µl Leibovitz L15 medium were implanted into the backside of 4-week-old female nude mice to assess their ability to initiate tumor xenografts. Tumors were measured weekly and their volume calculated as length × width × width/2.

### miR-27b Therapy

SW620 cells (5×10^6^) were injected into the backside of 4-week-old female NOD/SCID mice, all of which developed tumors in 1 week with a volume of ∼200 mm^3^. Five mice were randomly assigned to each of the negative control (NC) and miR-27b groups. All mice were intratumorally injected with cholesterol-conjugated mimics (1 OD mimics/time/mouse) (GenePharma Tech, Shanghai, China) twice per week and tumors measured every 4 days. The mice were euthanized by cervical dislocation 5 weeks after the tenth injection, and transplantable tumors were isolated and assessed.

### Immunofluorescence Assay

All xenograft tissues were formalin-fixed and paraffin-embedded for sectioning on a Leica microtome. Four-micron sections were prepared and antigen retrieval performed by boiling the samples for 15 min at 100°C in 10 mmol/l sodium citrate. Endogenous peroxidase activity was blocked with 0.3% hydrogen peroxidase for 20 min, and samples were blocked with PBS containing 1% FBS. Polyclonal rabbit anti-mouse CD31 IgG (abcam, Cambridge, MA, USA) was diluted 1∶200 and added as the primary antibody; samples were then incubated at 4°C overnight. Samples were subsequently incubated with the appropriate secondary antibody conjugated to fluorescein isothiocyanate (FITC) (Multisciences Biotech, Hangzhou, China).

### Luciferase Assay

The pmirGLO Dual-Luciferase miRNA Target Expression vector (Promega) contained the VEGFC mRNA 3′UTR of the miR-27b target site or a mutated miR-27b target site (see File S1). The pRL-TK plasmids (Promega) were co-transfected into 293T cells with either the negative control mimic (5′-UUCUCCGAACGUGUCACGUTT-3′), miR-27b mimic (5′-UUCACAGUGGCUAAGUUCUGC-3′), or anti-miR-27b mimic (5′-GCAGAACUUAGCCACUGUGAA-3′) (GenePharma Tech, Shanghai, China) using Lipofectamine 2000 (Invitrogen). The ratio of firefly to *Renilla* luciferase activity was determined using Dual Luciferase Reporter Assay System (Promega) 48 h after transfection in a luminometer.

### Western Blotting

Total protein was extracted from cells lysed with the M-PER Mammalian Protein Extraction Reagent (Thermo) supplemented with the cocktail of protease inhibitors (Sigma). After blocking with 5% non-fat milk in Tris-buffered saline with Tween-20 (TBST) for 60 min, the membrane was incubated with the primary antibodies anti-human-VEGFC (Cell signaling technology, Danvers, MA, USA) (1∶2000) or anti-human GAPDH (KangChen, Shanghai, China) dissolved in 5% bovine serum albumin in TBST overnight at 4°C.

### Clinical CRC Sample Analysis

Specimens were collected between 2008.1 and 2010.12 at the Second Affiliated Hospital, Zhejiang University School of Medicine and confirmed pathologically. Tumors were staged using the International Union Against Cancer (UICC) tumor staging system (Table S1).

### Statistical Analysis

Data are presented as the means ± standard error of the mean (SEM). The qPCR results from paired clinical samples were analyzed by a two-tail paired Student’s *t*-test and the other results by a two-tail unpaired Student’s *t*-test. *P* values <0.05 indicated statistical significance.

***Other methods***, including cell sorting, plasmid construction, establishment of miR-27b, anti-miR-27b, or VEGFC knockdown (VEGFC-shRNA anti-miR-27b stable SW620 cells), ELISA, hematoxylin and eosin (HE) staining, and methylation-specific polymerase chain reactions (MSP), are described in File S1.

## Results

### miR-27b Levels Decrease both in Colorectal CSCs and most Cancer Tissues

**Figure 1:**
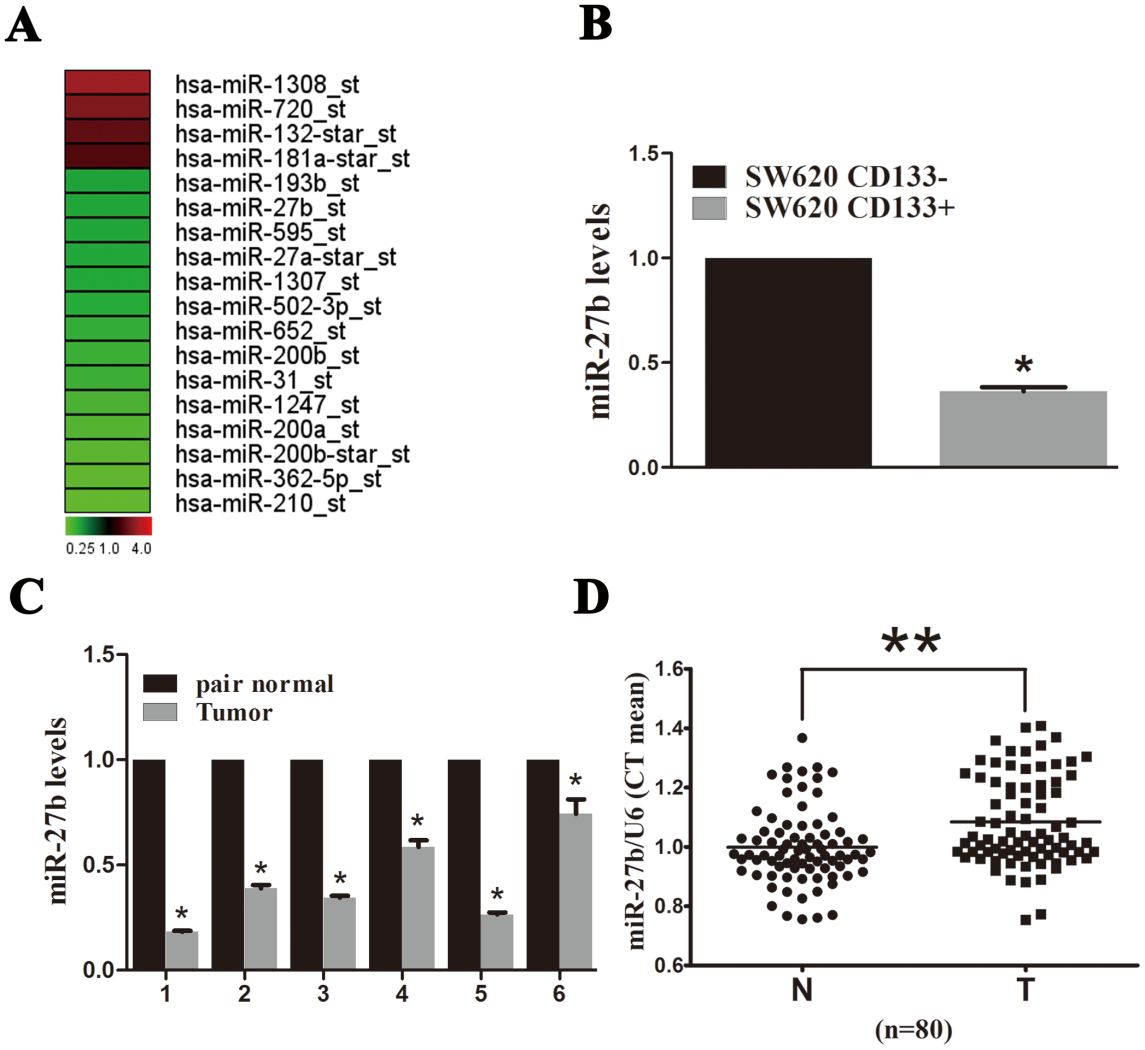
miR-27b expression in cancer stem cells (CSCs) and colorectal cancer (CRC) tumor tissue. (A) Differentially expressed miRNA in CD133^+^ and CD133^−^ cells. Red denotes high and green denotes low levels of expression. (B) miR-27b expression in CD133^+^ and CD133^−^ cells was assessed by qPCR. The y-axis indicates fold change. (C) miR-27b expression assessed by qPCR in fresh CRC tissues compared to adjacent normal tissues from six patients. The y-axis indicates fold change. (D) miR-27b expression assessed by qPCR in 80 paired paraffin-embedded CRC and adjacent normal tissues. miR-27b levels were normalized to U6 and expressed in terms of the threshold cycle (C_t_) ratio. Error bars represent the means ± SEM, *P<0.05, **P<0.01.

CSCs play a crucial role in carcinogenesis and are associated with recurrence, metastasis and therapy resistance [20]–[22]. A number of surface markers can be used for CSCs sorting, including CD24, CD44, CD166 and CD133 [22]. Of these, CD133 is a good CSCs marker of CRC [5], [22]–[25]. We observed CSCs properties in CD133^+^ SW620 cells (Figure S1) and assessed miRNA expression profiles in CD133^+^ and CD133^−^ cells to identify miRNAs involved in tumor progression. Microarray analysis detected four up-regulated (miR-1308, miR-720, miR-132-star and miR-181a-star) and 14 down-regulated (miR-27b, miR-193b, miR-595, miR-27a-star, miR-1307, miR-502-3p, miR-652, miR-200b, miR-31, miR-1247, miR-200a, miR-200b-star, miR-362-5p, miR-210) miRNAs in CD133^+^ cells (Figure 1A). When these results were combined with previous miRNA microarray data (data not shown), only miR-27b expression differed. This was confirmed by qPCR, which demonstrated a 2.77-fold change in miR-27b expression in CD133^+^ *versus* CD133^−^ cells (Figure 1B).

We also measured miR-27b expression in CRC tissue samples. In the limited number of available fresh tissue samples (n = 6), miR-27b expression was down-regulated 1.5–5.5-fold in CRC tissues compared to adjacent normal tissues (Figure 1C). In a larger number of paired paraffin-embedded tissues, the miR-27b to U6 threshold cycle (C_t_) value ratios were significantly higher in tumor tissues, indicating lower miR-27b expression in CRC (Figure 1D). Actually, the qPCR data of 80 paired paraffin-embedded CRC and adjacent normal tissues showed that miR-27b expression decreased in 60% CRC compared to 15% elevated. miR-27b has recently been reported to be a tumor suppressor in neuroblastoma; thus we focused the remainder of our studies on determining the biological functions and regulatory mechanisms of miR-27b in CRC.

### miR-27b Inhibits Tumor Growth and Angiogenesis in CRC

**Figure 2:**
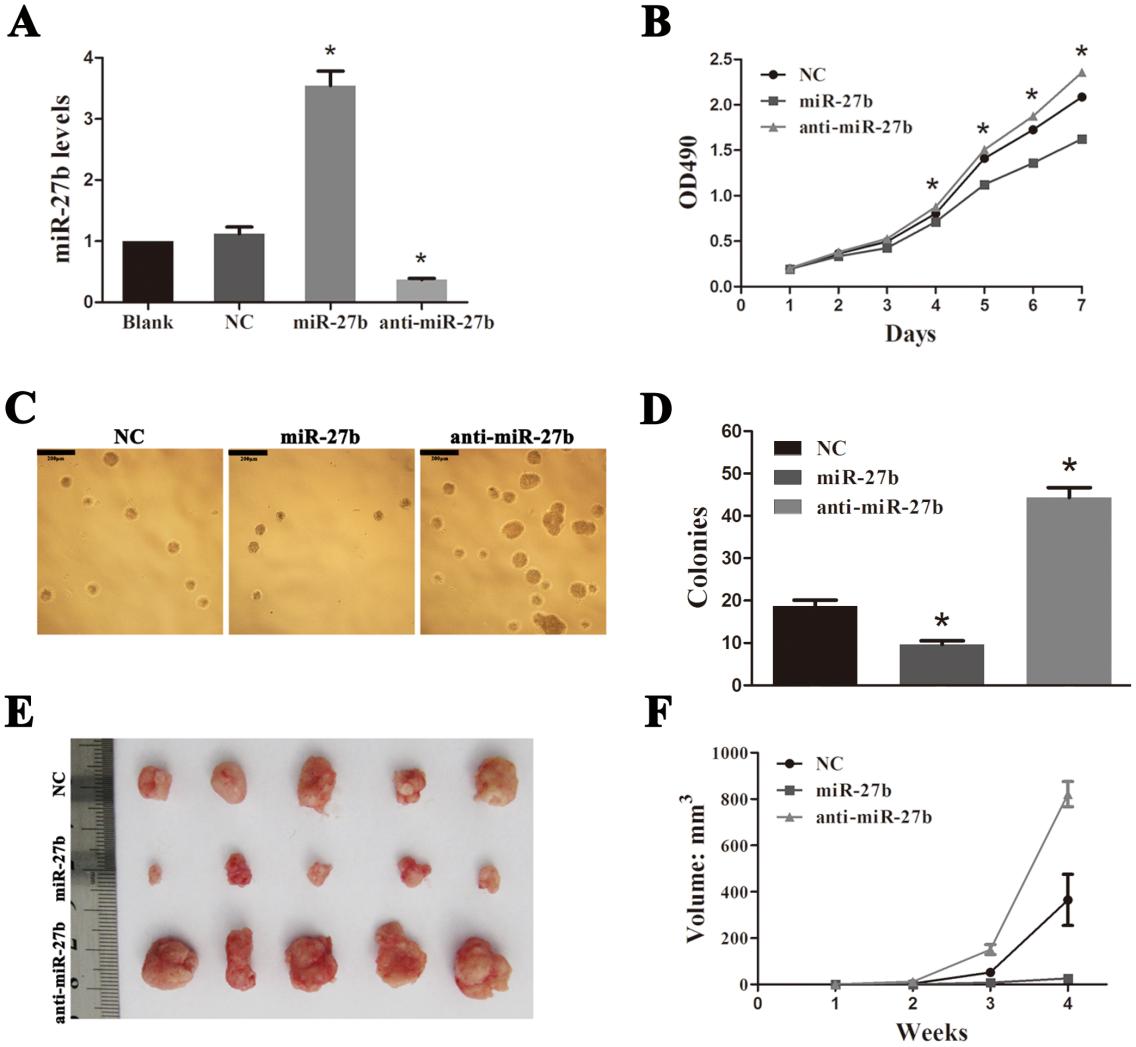
miR-27b inhibits tumor growth in colorectal cancer (CRC). (A) miR-27b expression in negative control (NC), miR-27b and anti-miR-27b SW620 cell lines were assessed by qPCR. The y-axis indicates fold change. (B) Cell proliferation rate detected by measuring the absorbance at 490 nm in an MTS assay. (C and D) Results of a soft-agar colony assay. Colonies were visualized by microscopy after 2 weeks of incubation. Colonies containing >20 cells were counted. Scale bars = 200 µm. (E) Results from a tumorigenesis assay. A representative image of xenograft tumors in nude mice injected subcutaneously with 1×10^6^ CRC cells. (F) Comparison of xenograft formation *in vivo*. Tumor volumes were measured each week. Error bars represent the means ± SEM, *P<0.05.

We established both miR-27b and anti-miR-27b SW620 stable cell lines (see File S1) to study the biological functions of miR-27b (Figure 2A) by determining proliferation and colony formation *in vitro* and tumorigenesis *in vivo*. We found that overexpression of miR-27b repressed cell proliferation, whereas inhibiting miR-27b through stable expression of an anti-miR-27b sponge promoted cell proliferation (Figure 2B). A soft-agar colony assay indicated that increased miR-27b expression significantly prohibited colony formation, known as self-renewal, whereas anti-miR-27b cells formed larger and greater numbers of spheres than the negative control (Figure 2C and D). More importantly, in a tumorigenesis assay initiated by subcutaneous injection of 1×10^6^ CRC cells, we found that miR-27b could strong suppress tumor growth, while anti-miR-27b promoted growth (Figure 2E and F).

**Figure 3:**
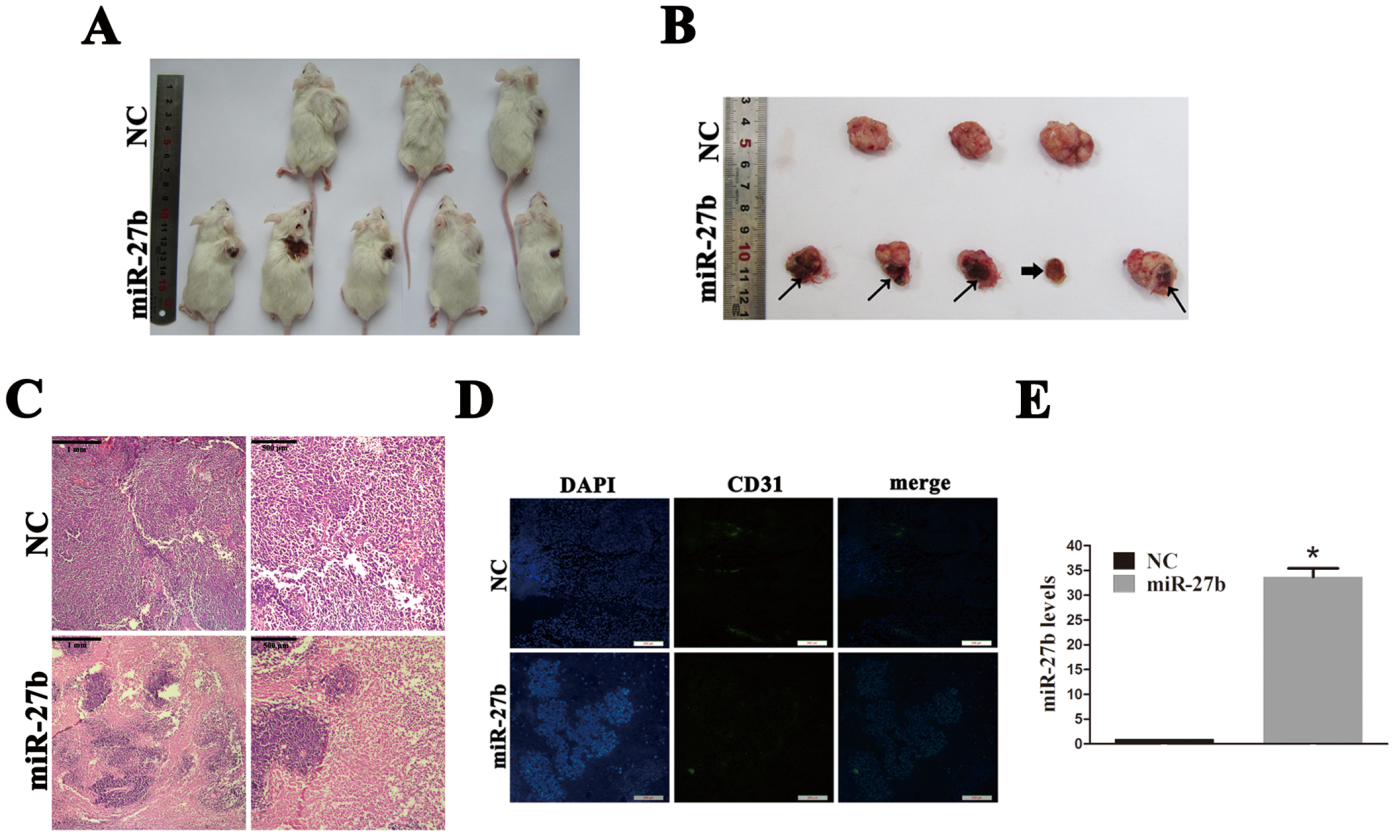
miR-27b has anti-tumor and angiogenesis effects *in vivo*. (A and B) Colorectal cancer (CRC) bearing NOD/SCID mice were intratumorally injected with cholesterol-conjugated negative control (NC) or miR-27b mimics. Scabs were observed in four tumors from the miR-27b group (fine arrow). One tumor from the miR-27b group disappeared completely with only a scab remaining (thick arrow). (C) Hematoxylin and eosin (HE) staining of xenograft tissues showing necrotic areas in the miR-27b group. (D) A representative immunofluorescence assay showing CD31 protein in xenograft tissues from NC and miR-27b (n = 3). Scale bars = 200 µm. (E) miR-27b expression in xenografts from NC and miR-27b mimics was assessed by qPCR. Error bars represent the means ± SEM, *P<0.05.

We further investigated the anti-tumor effect of miR-27b *in vivo* in a human CRC-bearing mouse model. The mice were randomly assigned into the negative control (NC) or miR-27b groups, with five mice per group. The cholesterol-conjugated NC or miR-27b mimics were injected into the tumors. Two mice in the NC group died after 4 weeks’ treatment; however, the cause of death was not determined. In the miR-27b group, one xenograft disappeared after 4 weeks of treatment (Figure 3A and B), while the other four xenografts were soft to the touch and severe tumor necrosis was observed upon pathological examination (Figure 3C). Immunofluorescence assays revealed that xenografts in the miR-27b group had less capillary blood vessels than those in the NC group (Figure 3D), and qPCR results confirmed that miR-27b levels were elevated significantly in miR-27b xenografts (Figure 3E). All of these findings support a tumor suppressive role for miR-27b in CRC and suggest its potential as an anti-CRC drug.

### VEGFC is a Novel Target of miR-27b in CRC

**Figure 4:**
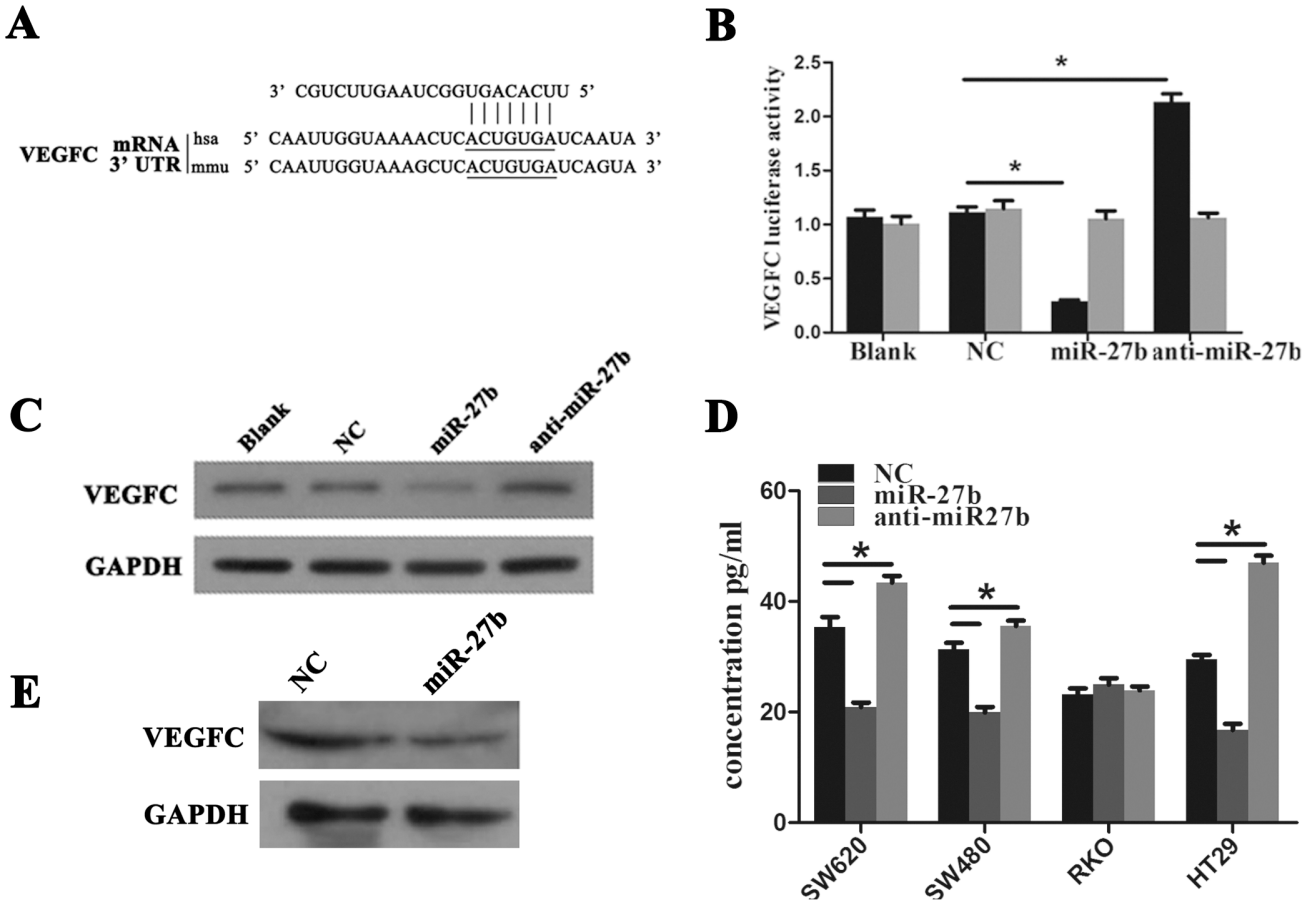
Vascular endothelial growth factor C (VEGFC) is a novel target of miR-27b in colorectal cancer (CRC). (A) VEGFC is predicted as a novel target of miR-27b. (B) 293T cells were co-transfected with empty pmirGLO Dual-Luciferase reporter plasmids or VEGFC 3′UTR firefly luciferase reporter plasmids and pRL-TK-luciferase plasmids, together with miR-27b mimics or anti-miR-27b. After 48 h, firefly luciferase activity was measured and normalized to that of Renilla luciferase. (C) CRC cells were transfected with NC, miR-27b or anti-miR-27b mimics and expression of VEGFC was detected by western blotting. (D) CRC cells were transfected with NC, miR-27b or anti-miR-27b mimics and VEGFC in culture medium was detected by ELISA. (E) VEGFC protein in xenografts from negative control (NC) and miR-27b mimics was detected by western blotting. Error bars represent the means ± SEM, *P<0.05.

miRNAs function primarily as mediators of gene silencing. Targets of miR-27b in CRC were subsequently analyzed using data predicted from the TargetScan database (www.targetscan.org). Hundreds of predicted miR-27b targets were subjected to further enrichment analysis of cell signaling pathways using the Kyoto Encyclopedia of Genes and Genomes (KEGG) pathway database (www.genome.jp/kegg/). Using this approach, miR-27b was predicted to target cancer-related signaling pathways including VEGF, Wnt and the mitogen-activated protein kinase (MAPK). Ultimately, we focused on VEGF signaling since Wnt and MAPK were not obviously affected in CRC (data not shown). We further identified VEGFC as a functional downstream target of miR-27b. The VEGFC 3′UTR contains highly conserved miR-27b binding sites (Figure 4A) that are responsive to miR-27b in a dual luciferase reporter assay. We found that the activity of a luciferase reporter containing the VEGFC 3′UTR decreased by ∼70% upon co-transfection with the miR-27b mimic, but increased by ∼100% upon co-transfection with the anti-miR-27b mimic. Moreover, no change was identified upon co-transfection of the mutant reporter plasmid with either miR-27b or anti-miR-27b mimics (Figure 4B). VEGFC protein levels were also decreased in cells and culture medium upon transfection with an miR-27b mimic, while VEGFC levels they increased upon transfection of an anti-miR-27b mimic (Figure 4C and D). *In vivo*, VEGFC protein levels were lower in miR-27b xenografts compared to in the NC group (Figure 4E).

### VEGFC Plays the Tumor-promoting Role in CRC

**Figure 5:**
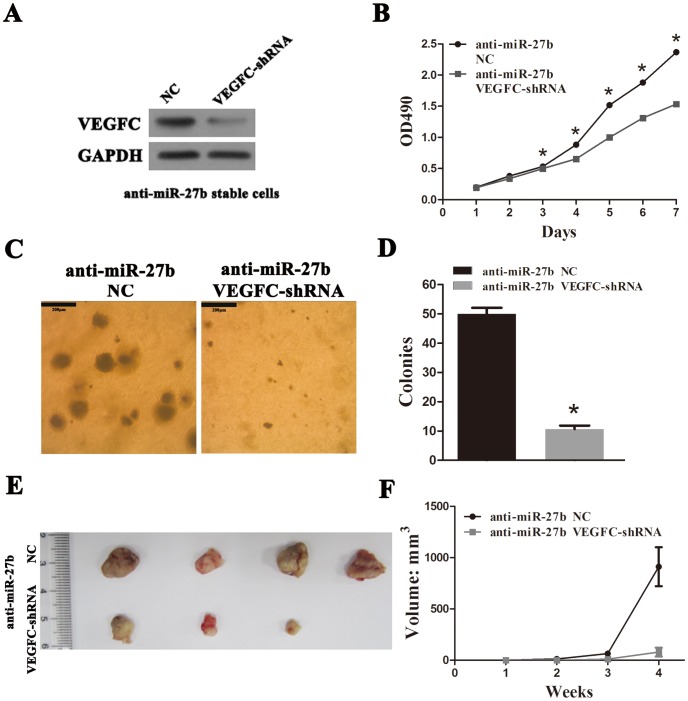
Vascular endothelial growth factor C (VEGFC) plays a tumor-promoting role in colorectal cancer (CRC). (A) VEGFC knockdown in anti-miR-27b stable cells was confirmed by western blotting. (B) Cell proliferation rate was determined by measuring the absorbance at 490 nm in a MTS assay. (C and D) Results of a soft-agar colony assay. Colonies were visualized by microscopy after 2 weeks of incubation. Colonies containing >20 cells were counted. Scale bars = 200 µm. (E) Results of a tumorigenesis assay. A representative image of xenograft tumors in nude mice subcutaneously injected with 1×10^6^ CRC cells. (F) Comparison of xenograft formation *in vivo*. Tumor volumes were measured each week. Error bars represent the means ± SEM, *P<0.05.

Many studies have reported that VEGFC correlates with tumor growth and metastasis in a variety of cancers, including CRC [16]–[18]. We established VEGFC-knockdown anti-miR-27b SW620 cells through expression of an inhibitory shRNA (see File S1) (Figure 5A). VEGFC-knockdown repressed cell proliferation compared to the NC cells (Figure 5B), significantly inhibited colony formation (Figure 5C and D) and reduced tumor growth (Figure 5E and F). Collectively, these observations strongly suggest that VEGFC plays role in stimulating proliferation and promoting tumorigenesis in CRC. Although there are a set of predicted miR-27b targets, VEGFC as a functional downstream target of miR-27b can be fully confirmed in our experiments.

### DNA Hypermethylation Reduces miR-27b Expression

**Figure 6:**
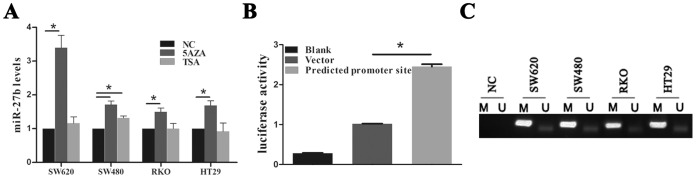
DNA hypermethylation is associated with decreased miR-27b expression. (A) The levels of miR-27b expression in colorectal cancer (CRC) cell lines were determined by qPCR after treatment with 5AZA or TSA for 3 days. (B) Results of luciferase activity assays following transfection with the predicted miR-27b promoter normalized to pRL-TK Renilla luciferase. (C) MSP analysis of the miR-27b CpG island in a set of CRC cell lines. Bands in the ‘M’ lanes are PCR products obtained using methylation-specific primers and those in the ‘U’ lanes are products obtained using unmethylated-specific primers.

Both transcriptional and epigenetic pathways regulate gene expression. Epigenetic mechanisms include DNA methylation, histone acetylation and non-coding RNAs [26]; silencing of some miRNAs is associated with CpG island hypermethylation in a variety of cancers [27]–[29]. To determine whether epigenetic mechanisms mediated miR-27b function, we cultured cells in the presence of the histone deacetylase inhibitor trichostatin A (TSA) (Sangon Biotech, Shanghai, China) or the methyltransferase inhibitor 5-aza-dC (5AZA) (Calbiochem, San Diego, CA, USA). miR-27b levels were unchanged in cells cultured with 1 nmol/ml TSA for 3 days. However, treatment with 5 nmol/ml 5AZA markedly elevated miR-27b expression (Figure 6A). These results suggest that DNA hypermethylation plays an important role in regulation of miR-27b. The predicted promoter site of miR-27b in chromosome 9 was cloned into a luciferase vector (see File S1) and verified using luciferase assays (Figure 6B). MSP results indicated miR-27b CpG island hypermethylation in several CRC cell lines (Figure 6C).

## Discussion

The CSCs hypothesis has been proven in a wide variety of solid tumors [20], [21], and the current literature is focused on the role of miRNAs in human cancer. miRNAs are deemed to have widespread regulatory activity in a broad range of developmental processes and are implicated in diverse diseases, including cancer [8].

We sought to investigate the function of miRNAs in CRC. We hypothesized that the molecular differences between CSCs and differentiated cancer cells may identify the key molecule responsible for tumor growth and progression. Both *in vitro* and *in vivo* investigations determined that CD133^+^ cells in CRC could be classified as CSCs-like cells based on their stem cell properties. This CSCs model was used to screen and identify 18 differentially regulated miRNAs. miR-27b was the only miRNA identified repeatedly in these experiments; no information regarding the role of this miRNA in CRC has been reported. We found that miR-27b did not affect CRC stem cell differentiation by altering expression of the stem-cell associated genes *Nanog, Oct4, Sox2, Bmi1* (data not shown). Further study showed decreased miR-27b expression in most CRC tissues.

We next investigated the function of miR-27b in CRC and demonstrated that it could significantly repress self-renewal *in vitro* and tumorigenicity *in vivo*. Moreover, we identified VEGFC as a functional downstream target of miR-27b using several methods. To our knowledge, this is the first study to report the specific function and a novel functional target of miR-27b in CRC. VEGFC belongs to the platelet-derived growth factor family and its expression correlates significantly with poorer histologic grade, lymphatic invasion and venous invasion [16]–[18], [30], and recent evidence suggests it has an important role in angiogenesis. [19], [31] Several recent studies report that autocrine regulation of cancer cells migration via VEGFC/VEGFRs is an important inducer of tumor cell proliferation, invasion and metastasis. [30] There is also emerging evidence supporting a putative role for miRNAs as tumor suppressors or oncogenes that could lead to targeted cancer treatment strategies [32], [33]. miR-34a has potent anti-tumor effects in prostate tumors and may represent a therapeutic agent for prostate cancer [11]. Intratumoral injection of cholesterol-conjugated miR-199a/b-3p mimics inhibited tumor growth and reduced serum AFP levels in hepatocellular carcinoma [34]. Malignant cells are dependent on aberrant miRNA expression; these small RNAs provide important opportunities for the development of future miRNA-based therapies [30], [35], [36]. Due to the serious side-effects of traditional chemotherapy, research on other methods for CRC treatment, such as gene therapy, is appealing.

Tumor angiogenesis is critical for tumor growth and maintenance, and many studies have demonstrated that angiogenesis inhibitors may provide a significant therapeutic advantage [37], [38]. Here we report that severe necrosis was observed in xenografts of miR-27b mimics, which also developed fewer capillary blood vessels than the NC group, and in one xenograft completely disappeared with only a scab remaining. These data demonstrate the anti-tumor effect of miR-27b *in vitro* and *in vivo*, suggesting miR-27b to be a promising target for CRC treatment after the efficacy and safety of gene therapy have been determined.

The mechanisms involved in the regulation of transcription are varied, and while those underlying miRNA dysregulation in cancer are not yet fully understood, miRNA-mediated promoter hypermethylation has been identified in the majority of tumors. We found that miR-27b mediated gene silencing in CRC was attributable to reversible hypermethylation of CpG islands and not histone acetylation.

The growth of blood vessels is essential for cancer growth and repair. Recent evidence indicated that tumor angiogenesis might be induced by CSCs due to angiogenic factor expression in the tumor microenvironment [37], [39]. Anti-angiogenesis therapy targeting VEGF can deplete the tumor vasculature and ablate self-renewing CSCs [37], [40]. Our data demonstrate that miR-27b originates in CSCs from CRC and acts as an important tumor suppressor and angiogenic factor by targeting VEGFC. Further study of CSCs or angiogenesis would facilitate the development of novel anticancer therapeutic strategies. miRNA-based therapeutic strategies may also result in improved management of tumors in the not-too-distant future [41], [42]. These results not only allow for a better understanding of the mechanisms regulating CRC cells but also facilitate the gradual development of more effective cancer therapies.

## Supporting Information

Figure S1 **CD133+ SW620 cells exhibit characteristics of CSCs** ***in vitro*** **and** ***in vivo*** **.** (A) Flow cytometry dot plot showing the distribution of CD133^+^ cells in the CRC cell line, SW620. (B and C) Results of a soft-agar colony assay. Colonies were visualized by microscopy after 2 weeks of incubation and those containing >20 cells were counted. Scale bars = 200 µm. (D) Results from a tumorigenesis assay. A representative image of xenograft tumors in nude mice that were injected subcutaneously with 5×10^4^ CD133^−^ or CD133^+^ SW620 cells. (E) Comparison of xenograft formation *in vivo*. Tumor volumes were measured weekly. Error bars represent the means ± SEM, *P<0.05.
(TIF)

File S1 (DOC)

Table S1 **Clinicopathological features of CRC patients.**
(DOC)
