# Supplementary figures and images for: miRNA-27b Targets Vascular Endothelial Growth Factor C to Inhibit Tumor Progression and Angiogenesis in Colorectal Cancer
Source: PLoS One. 2013 Apr 12;8(4):e60687. doi: 10.1371/journal.pone.0060687 (PMC3625233; doi:10.1371/journal.pone.0060687)

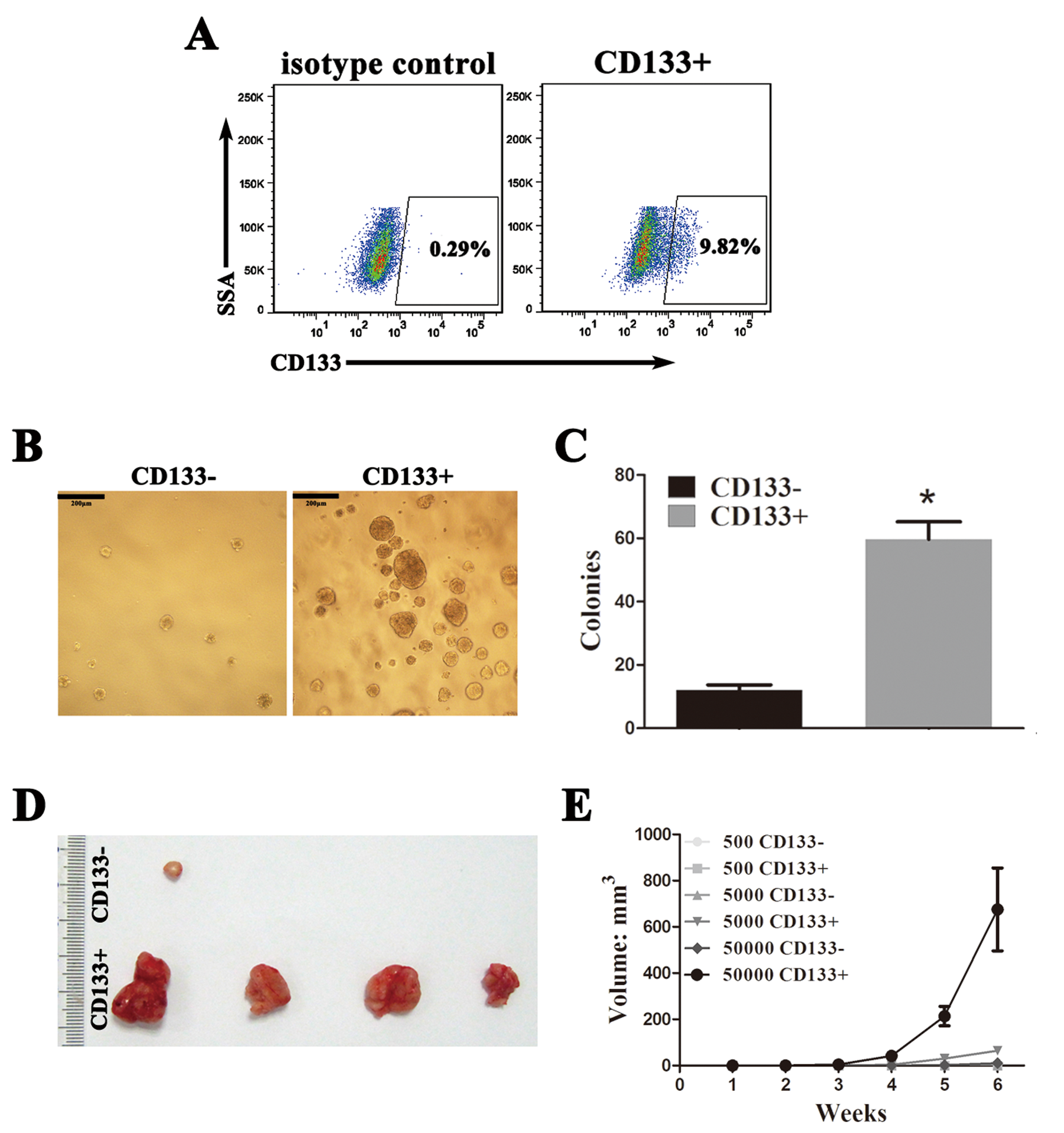

Supplement: Figure S1 — CD133+ SW620 cells exhibit characteristics of CSCs in vitro and in vivo . (A) Flow cytometry dot plot showing the distribution of CD133+ cells in the CRC cell line, SW620. (B and C) Results of a soft-agar colony assay. Colonies were visualized by microscopy after 2 weeks of incubation and those containing >20 cells were counted. Scale bars = 200 µm. (D) Results from a tumorigenesis assay. A representative image of xenograft tumors in nude mice that were injected subcutaneously with 5×10^4^ CD133− or CD133+ SW620 cells. (E) Comparison of xenograft formation in vivo. Tumor volumes were measured weekly. Error bars represent the means ± SEM, *P<0.05. (TIF) [file pone.0060687.s001.tif]
