# Supplementary material for: miRNA-27b Targets Vascular Endothelial Growth Factor C to Inhibit Tumor Progression and Angiogenesis in Colorectal Cancer
Source: PLoS One. 2013 Apr 12;8(4):e60687. doi: 10.1371/journal.pone.0060687 (PMC3625233; doi:10.1371/journal.pone.0060687)
Supplement: File S1 — (DOC) [file pone.0060687.s002.doc]

**Supporting Information**

***Flow cytometry***

SW620 cells (up to 2 × 10^7^) were labeled in phosphate-buffered saline (PBS) containing 2% FBS and an anti-human monoclonal CD133 antibody conjugated with R-phycoerythrin (Miltenyi Biotec GmbH, Bergisch Gladbach, Germany) for 10 min in the dark at 4°C. A blank control and mouse IgG_1_ R-phycoerythrin (Miltenyi Biotec GmbH) was used as an isotype control. Cells labeled with the CD133 marker were assessed using a FACSCanto II flow cytometer (BD Biosciences, San Diego, CA, USA) before sorting.

***Plasmid construction***

The 3’UTR of VEGFC containing the miR-27b target site (5’-AACTCACTGTGATCAATA-3’) or a mutant 3’UTR of VEGFC (5’-AACTCGTCACGATCAATA-3’) were cloned into the *Sac*I/*Xho*I site of the pmirGLO Dual-Luciferase miRNA Target Expression Vector (Promega). The predicted promoter site of miR-27b (chr9:96,885,608-96,886,897) was cloned into the *Kpn*I/*Hind*III site of pGL3-Enhancer Vector (Promega) using the following PCR primers: 5’-AATTATAGGTACCCACCTGGAGCACCCACAGCACACACACCACGCAGAGTACACGCG-‘3; 5’CTATAATGAAGCTTGGACTGGTTGGGGGGCAGGCTTGCAACCCCTGGATGCATCTGGA-‘3

***Establishment of miR-27b*** ***or*** ***anti-miR-27b stable cells***

The miR-27b or anti-miR-27b lentiviral particles (PG-LV3-H1-miR-27b-Puro/PG-LV3-H1-miR-27b inhibitor-Puro) (GenePharma Tech, Shanghai, China) were transfected into SW620 cells. Cells were selected with 5 μg/ml puromycin (Sangon Biotech) 48 h after transfection. The efficiency of overexpression or down-regulation was verified by RT-PCR.

***Establishment of VEGFC-knockdown anti-miR-27b stable cells***

A VEGFC shRNA plasmid (Santa Cruz Biotechnology, CA, USA) was transfected into anti-miR-27b stable SW620 cells using Lipofectamine 2000 (Invitrogen) in accordance with the manufacturer’s instructions. Cells were selected with 5 μg/ml puromycin 48 h after transfection and the knockdown efficiency verified by RT-PCR.

***ELISA***

The VEGFC concentration was determined using a quantitative sandwich ELISA using Human VEGF-C Platinum ELISA kit (eBioscience, CA, USA).

***Hematoxylin and Eosin (HE) Staining***

All tissue samples were formalin-fixed and paraffin-embedded, and sectioned on a Leica microtome. Four micron sections were transferred to adhesive-coated slides, de-paraffinized with standard xylene and hydrated through a graded ethanol in water series, stained with HE, and covered with a coverslip.

***Methylation-specific polymerase chain reaction (MSP)***

DNA was extracted from cell lines by DNeasy Tissue Kit (Qiagen). MSP was performed under the following cycling conditions: 95°C for 4 min, then 40 cycles of 94°C, 25 s; 51°C, 25 s; 72°C, 30 s, followed by a final 5 min extension at 72°C. MSP products were separated electrophoretically on 2% agarose gels and visualized under ultraviolet light after staining with ethidium bromide.

The primers for methylated MSP (MMSP) and unmethylated MSP (UMSP) are as follows:

MMSP 5’-ACGTAGCGCGTATATTGTATG-‘3, 5’-CGTATACTATATACGCCGCG-‘3; UMSP, 5’-TATATGTATTATATGTAGTGTGTATATTGTATG-‘3; 5’-CCACATATACTATATACACCACAAATAC-‘3.
