# Supplementary material for: miRNA-27b Targets Vascular Endothelial Growth Factor C to Inhibit Tumor Progression and Angiogenesis in Colorectal Cancer
Source: PLoS One. 2013 Apr 12;8(4):e60687. doi: 10.1371/journal.pone.0060687 (PMC3625233; doi:10.1371/journal.pone.0060687)
Supplement: Table S1 — Clinicopathological features of CRC patients. (DOC) [file pone.0060687.s003.doc]

**Table S1**. Clinicopathological features of CRC patients.

| Characteristics | Number |
| --- | --- |
| Gender |  |
| Male | 48 |
| Female | 38 |
| Age (years) |  |
| Male | 60 (25–83) |
| Female | 64 (40–83) |
| Pathogenic Site |  |
| Rectum | 4 |
| Sigmoid colon | 29 |
| Right hemicolon | 27 |
| Left hemicolon | 26 |
| Pathological Type |  |
| Tubular adenocarcinoma | 37 |
| Papillary adenocarcinoma | 39 |
| Mucinous adenocarcinoma | 6 |
| Complex | 4 |
| Differentiation |  |
| Well | 23 |
| Moderate | 43 |
| Poor | 20 |
| Invasion depth |  |
| T1 | 1 |
| T2 | 1 |
| T3 | 8 |
| T4 | 31 |
| T5 | 45 |
| Lymph node metastasis |  |
| N0 | 46 |
| N1 | 24 |
| N2 | 16 |
| Distant metastasis |  |
| M0 (NM group) | 67 |
| M1 (M group) | 19 |
| TNM stage |  |
| I | 3 |
| II | 8 |
| III | 74 |
| IV | 1 |

TNM stage: tumor-node-metastasis stage.
